# Does concomitant meniscus repair and meniscectomy show different efficacy in anterior cruciate ligament reconstruction? A systematic review and meta-analysis

**DOI:** 10.1016/j.jot.2024.07.004

**Published:** 2024-07-25

**Authors:** Gyula Ferenc Szőcs, Szilárd Váncsa, Gergely Agócs, Péter Hegyi, Dóra Matis, Gergely Pánics, Zoltán Bejek, György Márk Hangody

**Affiliations:** a Department of Orthopaedic Surgery and Traumatology, Uzsoki Hospital, Budapest, Hungary; b Centre for Translational Medicine, Semmelweis University, Budapest, Hungary; c Institute for Translational Medicine, Szentágothai Research Centre, Medical School, University of Pécs, Pécs, Hungary; d Division of Pancreatic Diseases, Heart and Vascular Center, Semmelweis University, Budapest, Hungary; e Department of Biophysics and Radiation Biology, Semmelweis University, Budapest, Hungary; f Department of Orthopaedics, Semmelweis University, Budapest, Hungary

**Keywords:** Anterior cruciate ligament injury, Cartilage injury, Meniscal repair, Meniscectomy, Meniscus surgery, Sport injury

## Abstract

**Aims:**

Currently, it is advised to perform meniscal repair instead of meniscectomy in certain cases of primary anterior cruciate ligament reconstruction (ACLR). However, the level of evidence is low. Therefore, this study aimed to compare the effectiveness of meniscectomy and meniscus repair in addition to ACLR.

**Methods:**

The systematic search was conducted in three online databases (EMBASE, MEDLINE, and Cochrane) from inception until October 2021 for the literature on primary ACLR and concomitant meniscal surgery. Eligible studies compared the following outcomes between meniscal repair and meniscectomy groups: the Knee injury and Osteoarthritis Outcome Score (KOOS), Lysholm score, International Knee Documentation Committee (IKDC) score, and KT-arthrometer examinations. Lastly, we calculated pooled mean differences (MDs) with 95 % confidence intervals (CIs) from the change between pre- and post-intervention values.

**Results:**

Of 10,565 studies, 22 met the inclusion criteria, with a follow-up between 6 and 43 months. We found no difference when comparing the KOOS subscale changes—only in the KOOS pain subscale (MD = −1.6; CI: −2.48, −0.72). However, these results were not clinically significant. We analyzed the lateral and media meniscal injuries separately and concluded the same results regarding KOOS changes. We found no significant differences in the Lysholm score change (MD = −2.61; CI: −5.51, 0.29), changes in IKDC score (MD = 1.08; CI: −4.05, 6.21) or the change for the KT-arthrometer side-to-side difference (MD = −0.50; CI: −1.06, 0.06).

**Conclusion:**

Based on our result, we did not find a clinically significant difference between meniscus repair and meniscectomy during primary ACLR regarding patient-reported outcomes in a short-term follow-up.

**Translational potential:**

Our research supports the prompt integration of findings into clinical practice for treating meniscus injuries during ACL reconstruction. We recommend considering both meniscus repair and meniscectomy, as the available data indicate their effectiveness. Further studies are necessary to assess the long-term impacts, particularly on osteoarthritis, and to identify patient subgroups that may benefit most from each technique.

## Graphical abstract

**Figure:**
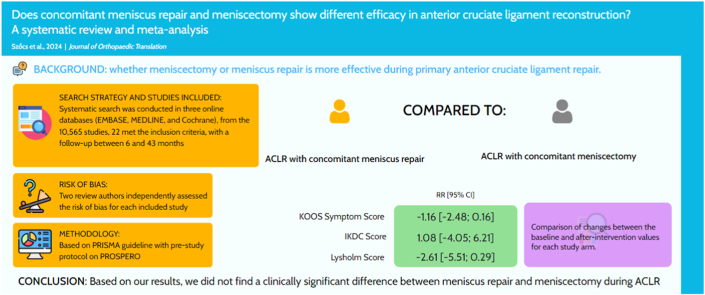

## 1 Introduction

Anterior cruciate ligament (ACL) injuries are among the most common musculoskeletal injuries, with 120,000 to 200,000 cases per year in the USA alone [1,2]. Most frequently, it results from non-contact injuries due to multi-plane and rotational forces [3,4]. Furthermore, damage to the ACL tends to occur in conjunction with multiligamentary or meniscal injury. The latter can go as high as 82 % in acute cases, with 44 % being lateral and 56 % being medial meniscal injuries [5].

Studies have shown that the main roles of menisci in the knee joint are to increase stability, distribute the axial load, absorb shock, and provide lubrication and nutrition inside the joint [6,7]. Therefore, during ACL reconstruction (ACLR), the two major surgical options for treating a torn meniscus are meniscectomy and meniscal repair.

Medical professionals are urging the increase of meniscal repairs, and conferences are calling for saving the meniscus. Even though the number of meniscus repairs has increased over the years, meniscectomy is still performed in 94 % of cases [8]. Unfortunately, menisci show poor healing rates due to their relative avascularity [9]. Therefore, the tear's localization and size can lead to irreparability [10]. In addition, the repair technique is associated with longer operating times, more surgical knowledge, and higher expenses. However, this is not entirely true in the setting of ACLR [11].

In cases of isolated meniscal tears, it is clear that meniscal repair can lead to better postoperative outcomes and lower rates of progression to knee osteoarthritis compared to meniscectomy [12,13]. However, a higher rate of reoperation is also associated with meniscal repair [14]. Studies comparing concomitant meniscectomy and meniscus repair in ACLR showed promising results for the International Knee Documentation Committee (IKDC) and Tegner scores in the early follow-up period. However, after 18 months, the scores decreased in the meniscectomy group [15]. It has also been recorded that meniscal repair performed with ACLR resulted in fewer reoperations [16]. However, there is no consensus in the literature on which intervention is favored with concurrent ACLR.

Therefore, our systematic review and meta-analysis aimed to compare the safety and effectiveness of meniscectomy and meniscus repair in addition to ACLR. We hypothesized that patients with meniscus repair would have better outcomes than meniscectomy patients.

## 2 Methods

We reported our systematic review and meta-analysis based on the recommendations of the PRISMA 2020 guideline [17] (see Supplementary Table 1), while we followed the Cochrane Handbook [18]. We registered the pre-study protocol on PROSPERO (registration number XXXXXXXXXX) and fully adhered to it.

### 2.1 Information sources and search strategy

We searched MEDLINE (via PubMed), Embase, and Cochrane Central Register of Controlled Trials (CENTRAL) databases for relevant publications. The search was performed from inception to October 27, 2021.

During the systematic search, we used the following search key ((meniscal OR meniscus) AND (repair OR fixation OR suture OR surgical OR surgery) AND (ACL or anterior cruciate ligament OR knee ligament OR cruciform ligament)). No language or any other filters were used during the search.

### 2.2 Selection process

The selection was performed by two independent review authors (Author1 and Author5) after duplicate references were removed, by title, abstract, and full text based on pre-discussed aspects. We used the Endnote v9.0 (Clarivate Analytics, Philadelphia, PA, USA) reference manager software for the selection process. We resolved disagreements during the selection by consensus; if consensus was not reached, a third independent review author (Author2) was involved in the decision.

In cases of overlapping populations, we selected the studies with the greatest number of participants.

### 2.3 Eligibility criteria

We used the PICO framework to formulate our research question. Eligible full-text articles reported on (P) patients with concomitant meniscal and ACL tears in the same knee joint while comparing (I) meniscus repair to (C) meniscectomy in addition to primary ACLR. Regarding the population, eligible studies included ACLR patients with mixed or either lateral or medial meniscal injury. We included all types of meniscal injuries. On the other hand, we did not have a pre-defined methodology for ACLR. The primary outcome of interest (O) was the comparison of clinical scores between the groups. Therefore, we included all the clinical outcomes in our synthesis, e.g., Lysholm, Knee Injury and Osteoarthritis Outcome (KOOS), and International Knee Documentation Committee (IKDC) scores, or comparison of sagittal instability of the knee joint (KT-arthrometer) [19].

We included randomized and non-randomized interventional studies. Exclusion criteria included: (1) ACLR revision surgery; (2) non-reconstructive ACL surgery; (3) conservative meniscal treatment; (4) studies reporting non-clinical outcomes; (5) multi-ligamentous injury, or (6) concomitant bony injuries. Moreover, we excluded conference abstracts, descriptive studies, reviews, and case reports.

### 2.4 Data collection process and data items

Two authors (Author 1 and Author 5) independently collected data from the eligible articles. In cases of disagreement, the decision was based on consensus or, if this was not reached, by involving a third author (Author 2).

We used a pre-defined data extraction table, and the following data were extracted: first author, year of publication, study population, study period, study site (country), study design, demographic data of the patients, time to surgery and follow-up time after ACLR and meniscal surgery, type of ACLR, specifics of meniscectomy and meniscal repair, pre- and postoperative data of the involved outcomes, and information for assessing the risk of bias in the studies.

The primary aim was to extract the mean and standard deviation (SD) of the changes between the baseline and after-intervention values for each study arm. If these were not directly available, the mean of change was calculated as the difference between the after-intervention and the baseline means. The SD of change was calculated using the after-intervention and baseline SDs and assuming 0 correlation based on the clinical expectation that the scores may only improve after intervention. Moreover, we also extracted the after-intervention values even if the baseline values were missing. In cases where the mean and the SD were missing but the median and the quartiles were reported, we estimated the mean and the SD using the equation of Luo et al. [20] for the former and the equation of Wan et al. [21] for the latter.

The score systems that were included in the meta-analysis (KOOS, IKDC, Lysholm) range between 0 and 100 points; based on previous literature, we considered the clinically significant difference to be over 8–10 point difference, we also relied on previous articles in the case of the results of the KT-arthrometer examinations [[22], [23], [24]].

### 2.5 Study risk of bias and level of evidence assessment

Two review authors independently assessed the risk of bias for each included study using the Risk Of Bias In Non-randomized Studies of Interventions (ROBINS-I) tool [25]. Disagreements were resolved by consensus. We used the Risk-of-bias VISualization (RobVis) web-based tool to visualize summary plots of the assessed domains [26].

We summarized the quality of evidence using the Grades of Recommendation, Assessment, Development, and Evaluation (GRADE Pro) tool [27]. To determine the level of evidence of the included studies we followed the Cochrane Handbook (Chapter 14.2) [18].

### 2.6 Synthesis methods

We performed a quantitative analysis in case of outcomes with at least three studies. Outcomes with fewer than three studies were included in the qualitative synthesis. We excluded studies from the meta-analysis if they reported data in an inappropriate format. However, we summarized these studies in a summary table.

All statistical analyses were made using the *meta* package (version 6.1.0) with R (R Core Team 2020, v4.0.3). We calculated pooled mean differences (MDs) with 95 % confidence intervals (CIs) between the study groups from after-intervention values or the changes between before- and after-intervention values of the outcome data. A random effects model was applied since we expected considerable between-study heterogeneity. To estimate the between-study variance (tau squared) the restricted maximum-likelihood (REML) method was used [28]. To estimate the CIs the Hartung‐Knapp method was used [29]. We used forest plots to represent pooled and individual study results.

When assessing the pooled effect size, a *p*-value <0.05 was considered statistically significant. *I*^2^ and χ^2^ tests were used to assess the statistical heterogeneity. We could not perform a publication bias analysis since fewer than ten studies were included in each pool.

A subgroup analysis was carried out for lateral and medial meniscus injuries.

## 3 Results

### 3.1 Search and selection

**Fig 1:**
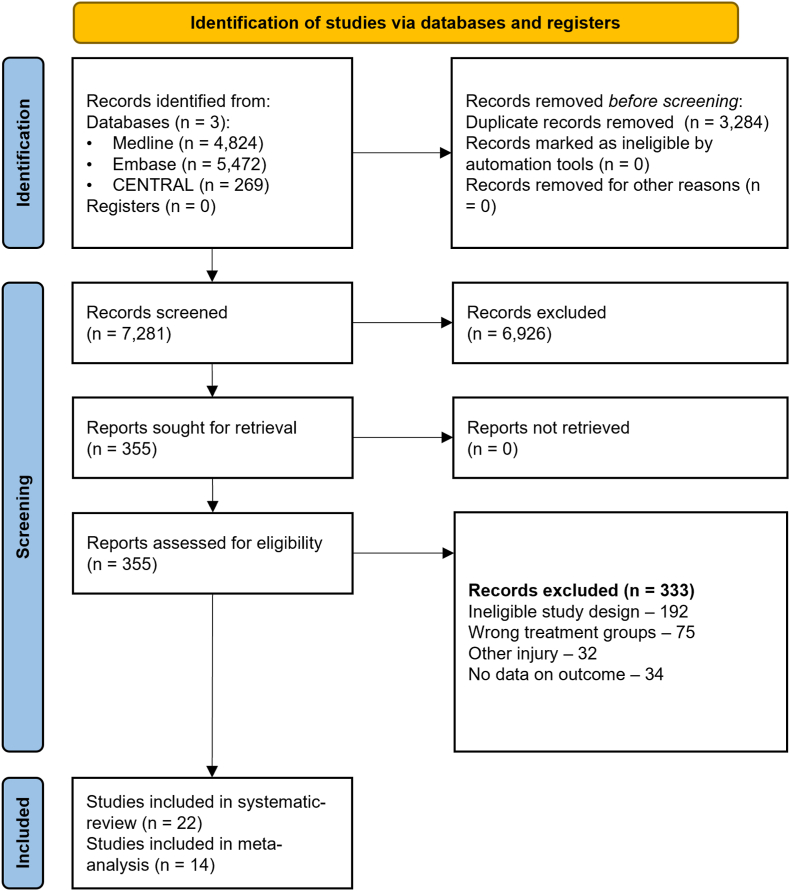
PRISMA 2020 flowchart representing the study selection process.

Altogether 10,565 studies were identified by our search, from which 22 full-text articles were eligible for our synthesis following the selection process described above [15,[30], [31], [32], [33], [34], [35], [36], [37], [38], [39], [40], [41], [42], [43], [44], [45], [46], [47], [48], [49], [50]]. We present the selection process details in Fig. 1. Out of the 22 articles, 14 articles were eligible for quantitative analysis [15,30,[32], [33], [34], [35], [36],^38^,^40^,^44^,[47], [48], [49], [50]].

### 3.2 Basic characteristics of included studies

The baseline characteristics of the included studies are detailed in Table 1 and Table 2. In Supplementary Table 2, we summarized the eligibility criteria of each included article. Regarding geographical localization, five studies originated from the USA, four from Sweden, two each from China, Singapore, Japan, and Turkey, and one article each from France, Norway, Greece, Ireland, and the United Kingdom. The type of meniscal injuries varied, including cleavage, beak and bucket handle tears, and ramp lesions. The meniscus repair technique used during the surgeries consisted of inside-out, outside-in, and all-inside, while the meniscectomy was either partial or total. The mean follow-up period after the concomitant ACLR and meniscal surgery ranged between 6 and 43 months.

**Table 1:** Basic characteristics of included studies in the meta-analysis.

Author (year)	Study site	Study type	Number of patients (I/C)	Meniscus injury	Meniscus repair description	Meniscectomy	Follow-up period(s) ‡	Outcome(s)
Balazs et al. (2020) [30]	USA	R	RAMP: 23/12 other: 5/23	RAMP or other meniscal lesions	all-inside	partial	1, 2 and 5 years	PRO score, SF12 PCS, SF12 MCS, **IKDC**, Marx activity scale
Casp et al. (2021) [32]	USA	R	Overall 71/44	NR	all-inside or inside-out	partial	5.9–6.1 months	IKDC, KOOS, Knee extension peak torque, Knee flexion peak torque, LSI: extension, LSI: flexion, Single-leg hop m/m, Triple hop m/m, 6-m timed hop, LSI: single hop, LSI: triple hop, LSI: 6-m timed hop
Cristiani et al. (2017) [33]	Sweden	R	MM: 207/559 LM: 153/593	medial or lateral	all-inside technique with the FasT-Fix, inside-out technique with PDS 0	partial	6 months	KT-1000 arthrometer
Cristiani et al. (2020) [34]	Sweden	R	MM: 198/653 LM: 142/658 BM: 30/181	medial, lateral or both menisci	all-inside technique with the FasT-Fix, inside-out technique with PDS 0	partial	1 and 2 years	KOOS
Dejour et al. (2020) [35]	France	P	MM: 25/87 LM: 16/26	medial or lateral	NR	resected	14.3 months	KOOS
Eken et al. (2020) [36]	Turkey	R	15/29	medial	NR	NR	3.6 years	Lysholm, IKDC, HSS knee score, Lachman, Pivot shift
Hoshino et al. (2021) [38]	Japan	P	139/30	medial and lateral	NR	resection	2 years	Lysholm, IKDC, KOOS, Lachman, KT-1000 (mm), Pivot shift, Medial JSW, Lateral JSW
LaPrade et al. (2015) [40]	Norway	R	MM: 318/898 LM: 111/647	medial or lateral	inside-out, all-inside	NR	2 years	KOOS
Lee et al. (2019) [15]	Singapore	R	17/22	medial or lateral	outside-in, all-inside	partial	19.4 and 27.6 months	IKDC, Tegner
Philips et al. (2018) [44]	Sweden	R	MM: 839/3552 LM: 423/3136	medial or lateral	NR	resection	2 years	KOOS, EQ-5D Index, EQ-5D VAS
Singh et al. (2017) [47]	Singapore	R	MM: 12/25 LM: 8/20	medial or lateral	all-inside	partial	3.5 years	Tegner, Lysholm
Svantesson et al. (2017) [48]	Sweden	R	MM: 225/773 LM: 179/663	medial or lateral	all-inside FasT-Fix, inside-out, outside-in	resection	6 and 12 months	KOOS, Lysholm
Wang et al. (2021) [49]	China	R	18/14	medial	fast-fix all inside	partial	24.8 months	IKDC, KOOS, kinematic, knee-degenartion
Yang et al. (2021) [50]	China	R	69/96	medial and lateral	outsied-in suture, all-inside FasT-Fix	partial	39.5 months	VAS, Lysholm, IKDC, Tegner, KT-2000 (mm)

**Table 2:** Basic characteristics of studies not included in the meta-anylisis

Author (year)	Study site	Study type	Number of patients (I/C)	ACL injury/meniscus injury	Meniscus repair description	Meniscectomy	Follow-up period(s)^a^	Outcome(s)
Byrne et al. (2021)^b^ [31]	Ireland	R	MM: 16/16 LM: 17/114	medial and/or lateral	NR	partial and total	9–11 months	IKDC, Marx score, ACL RSI, Strength and jump performance metrics, Return to play
Hatayama et al. (2020)^b^ [37]	Japan	R	24/32	medial	inside-out	partial	2 years	Lysholm, Pivotshift, Tegner
Kacmaz et al. (2021)^b^ [39]	Turkey	R	11/9	medial or lateral	all-inside Fast-Fix	partial	17 and 16 months	Lysholm, Tegner, KOOS, WOMAC, FJS-12 (total)
Lepley et al. (2014)^b^ [41]	USA	R	12/10	NR	NR	partial	7.5 and 7.6 months	Quad activation, Quad isokinetic, Quad isometric
Melton et al. (2011)^b^ [42]	UK	P	40/40	medial and/or lateral	inside-out suture	partial	7.7–12.6 years	IKDC, Lysholm, survival analysis
Michalitsis et al. (2016) [43] b	Greece	P	7/14	medial or lateral	all-inside FasT-Fix	partial	27.8 ± 4.8 months	Lysholm, Tegner, KOOS
Shelbourne (2003)^b^ [45]	USA	R	56/99	medial	inside-out	partial	6–8 years	Noyes score, overall/radiographic IKDC
Shelbourne (2004)^b^ [46]	USA	R	67/24	lateral	inside-out	partial	7–11.1 years	Noyes score, overall/radiographic IKDC

ACL-RSI: Anterior Cruciate Ligament Return to Sport after Injury; BM: both menisci; EQ-5D: EuroQol five–dimension scale questionnaire; FJS-12: forgotten joint score-12; HSS: Hospital for Special Surgery Knee-Rating Scale; IKDC: International Knee Documentation Committee; JSW: joint space width; KOOS: Knee Injury and Osteoarthritis Outcome Score; KT: KT knee arthrometer; LM: lateral meniscus; LSI: Limb Symmetry Index; NR: not reported; MM: medial meniscus; PRO score: Patient-reported outcome; SF12 MCS: short form survey 12 mental component score; SF12 PCS: short form survey 12 physical component score; USA: United States of America; VAS: visual analog scale; WOMAC: Western Ontario and McMaster Universities Arthritis Index;

a Parameters represented as mean or median

b Study included only in the systematic review

### 3.3 KOOS subscales did not differ significantly between the meniscectomy and meniscus repair groups

Five articles reported the score change from preoperative to postoperative in the five subscales of KOOS (2218 vs. 9769 patients), and seven reported the postoperative values (2330 vs. 9926 patients in the meniscus repair and meniscectomy groups, respectively).

**Fig 2:**
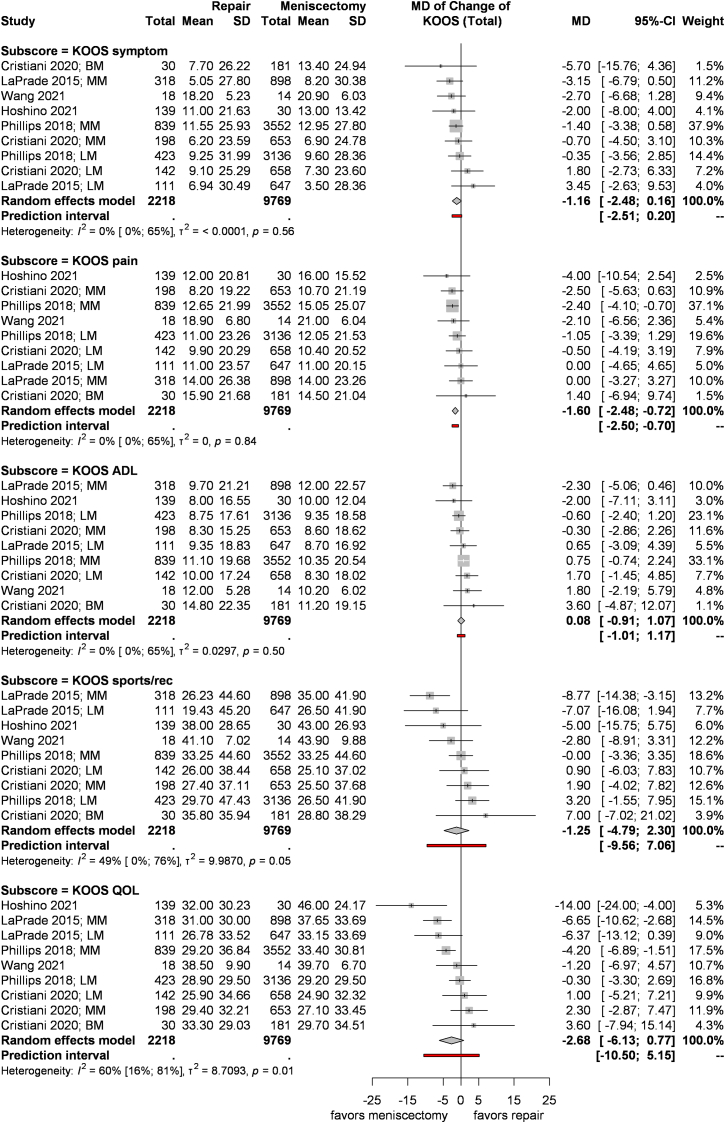
Forest plots representing the mean changes in the different KOOS subscales between meniscus repair and meniscectomy in addition to primary ACLR. ACLR: anterior cruciate ligament reconstruction, ADL: activities of daily living, CI: confidence interval, KOOS: Knee Injury and Osteoarthritis Outcome Score, LM: lateral meniscus, MD: mean difference, MM: medial meniscus, SD: standard deviation, QOL: quality of life.

We found significantly lower postoperative KOOS Symptom (MD = −1.71; CI: −2.86, −0.55; I^2^ = 25 %, p = 0.19; Supplementary Fig. 1) and KOOS Pain (MD = −1.2; CI: −2.13, −0.28; I^2^ = 32, p = 0.14; Supplementary Fig. 1) values in the meniscectomy group compared to the patients in the meniscus repair group. The difference in KOOS function in daily living (ADL), function in sport and recreation (Sport/Rec), and knee-related quality of life (QOL) did not differ between the groups. When comparing the change, we found significantly lower scores in the meniscectomy group only for the KOOS pain subscale (MD = −1.6; CI: −2.48, −0.72; I^2^ = 0 %, p = 0.84; Fig. 2). However, none of the resulting differences were clinically significant.

Similar KOOS improvement between meniscectomy and meniscus repair for lateral and medial meniscus injuries.

We analyzed the effectiveness of meniscectomy and meniscus repair separately for lateral and medial meniscal injuries. As a result, four articles reported separately on medial injuries and three studies on lateral meniscal injuries regarding KOOS.

**Fig 3:**
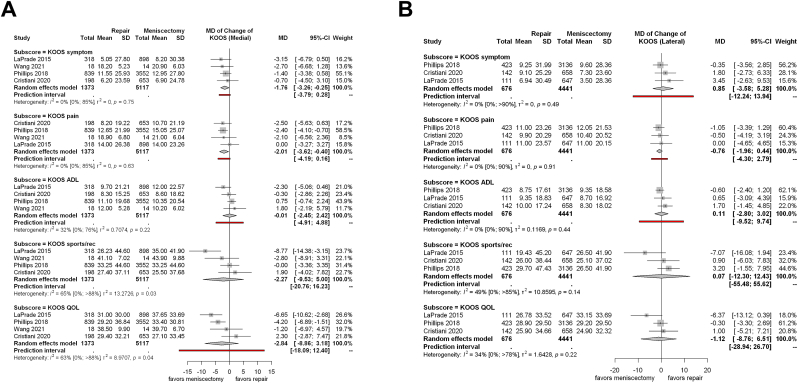
Forest plots representing the mean changes in the different KOOS subscales between meniscus repair and meniscectomy for (A) medial and (B) lateral meniscus injuries in addition to primary ACLR.

Considering the score changes of medial meniscal injuries, in the KOOS Symptom (MD = −1.76; CI: −3,26, −0,25; I^2^ = 0 %, p = 0.75; Fig. 3A) and Pain (MD = −2.01; CI: −3.62, −0.40; I^2^ = 0 %, p = 0.63; Fig. 3A) subscales there was a significantly lower increase in the meniscectomy group. The after-treatment scores showed the very same results (Supplementary Fig. 2A). In the case of lateral meniscal injuries, a significant difference could not be found either in the changes (Fig. 3B) or the follow-up data (Supplementary Fig. 2B). Moreover, the differences were not clinically significant for any of the scores.

### 3.4 IKDC and Lysholm scores did not differ between the meniscectomy and meniscus repair groups

**Fig 4:**
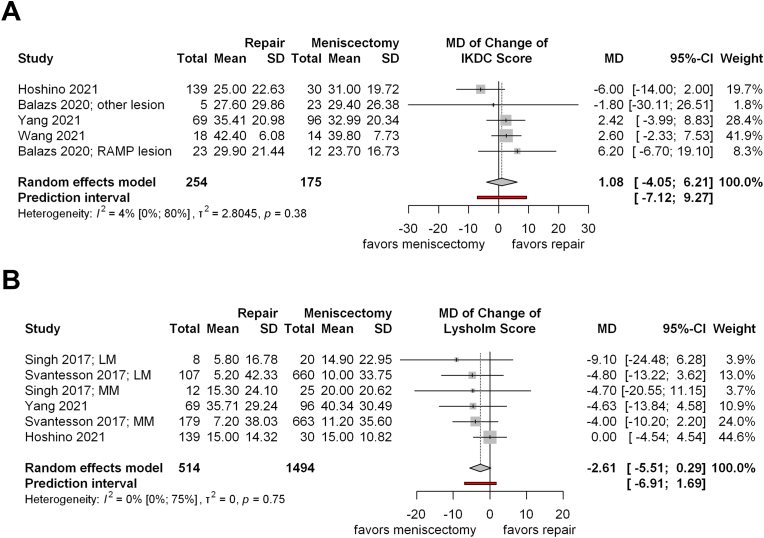
Forest plots representing the mean changes in (A) IKDC and (B) Lysholm score between meniscus repair and meniscectomy in addition to primary ACLR CI: confidence interval, IKDC: International Knee Documentation Committee, LM: lateral meniscus, MD: mean difference, MM: medial meniscus, SD: standard deviation,

On the other hand, four studies reported the Lysholm score changes. We found no significant differences between the meniscectomy and meniscus repair groups. However, the meniscus repair group tended to improve more (MD = −2.61; CI: −5.51, 0.29; I^2^ = 0 %, p = 0.75; Fig. 4A). Comparing the after-treatment scores, the result was the same (MD = −1.15; CI: −3.40, 1.11; I^2^ = 3 %, p = 0.39; Supplementary Fig. 3A).

Furthermore, four articles examined the changes in the IKDC score. The results favored the meniscal repair group, but no significant difference could be found (MD = 1.08; CI: −4.05, 6.21; I^2^ = 4 %, p = 0.38; Fig. 4B). Similarly, we did not find a significant difference in the follow-up values (MD = −1.23; CI: −4.63, 2.16; I^2^ = 50 %, p = 0.05; Supplementary Fig. 3B).

### 3.5 Anterior translation of the tibia examined by KT-arthrometer

Lastly, based on three articles, we compared the changes (MD = −0.50; CI: −1.06, 0.06; I^2^ = 0 %, p = 0.42; Supplementary Fig. 4A) and after-treatment results (MD = −0.41; CI: −0.97, 0.15; I^2^ = 49 %, p = 0.12; Supplementary Fig. 4B) of the anterior translation of the tibia between the groups. No significant difference could be seen in either analysis. However, both of them tended to favor meniscus repair.

### 3.6 Risk of bias assessment and level of evidence

The results of the risk of bias assessment are presented in Supplementary Table 3. Most of the studies presented a moderate risk of bias.

A summary of the quality of evidence can be found in Supplementary Tables 4–6. All outcomes resulted in a low quality of evidence due to the risk of bias, study type, and possibility of reporting bias.

## 4 Discussion

Our study did not find a significant difference between meniscectomy and meniscus repair in addition to ACLR regarding patient-reported outcomes (KOOS, IKDC, Lysholm) and anterior tibial translation measured by arthrometry. Both surgical interventions had the same effectiveness, improving the scores by roughly the same amount.

It is important to mention that there is a lack of randomized studies on this topic. One reason may be that not every type of meniscal tear can be repaired; it depends on the size, shape, and localization of the injury. On the other hand, magnetic resonance imaging does not have perfect specificity and sensitivity for meniscal ruptures, resulting in the decision on the used method being mainly intraoperative [51].

Based on current guidelines, there are no clear recommendations for concomitant meniscectomy or meniscus repairs in addition to ACLR. As mentioned in the 2022 revision of the Management of Anterior Cruciate Ligament Injuries published by the American Academy of Orthopedic Surgeons, the main reason behind this fact is the low quality of studies published on this topic. On the other hand, they highlight the need for long-term studies focusing on meniscus repair and the rates of osteoarthritis progression to determine this procedure's real value [52].

Most studies reported on short and mid-term outcomes (6 months–2 years). This short-term follow-up may prevent the chondroprotective effect of meniscal repair in addition to ACLR. In the case of isolated meniscal injuries, osteoarthritic progression was detectable up to three times more than in the meniscectomy group with an 8.8-year mean follow-up [53]. Also, a previous meta-analysis showed that meniscal resection combined with ACLR increased the risk of developing radiographic osteoarthritis compared to patients who underwent meniscectomy after a 10-year follow-up [54].

Of the included studies, the longest follow-up time was reported by Melton et al. with a median follow-up time of 10 years. In this study, patients undergoing ACLR combined with meniscal repair had a significantly higher IKDC than in the meniscectomy group (84.2 vs. 70.5, respectively; *p* = 0.008). However, compared to isolated ACL injuries, the meniscus repair group had lower IKDC values (88.3 vs. 84.2, respectively; *p* = 0.005). On the other hand, the same authors predicted a meniscal suture survival rate between 49 % and 89 % ten years after the initial surgery due to high loss to follow-up (35 %) [42].

It is known that meniscal repair helps the healing of the reconstructed ACL graft [55]. Yang et al. reported a 10.1 % failure rate of medial meniscus repair during ACLR. However, the surgical outcomes of ACLR were not affected, suggesting that even in the event of meniscus repair failure it can be beneficial because of graft protection. On the other hand, meniscus retears can be successfully treated by subsequent partial meniscectomy in patients with repair failure [50]. Cristiani et al. reported their reoperation rates of 12.4 % for MM repair, 7.2 % for LM repair, and 16.7 % for MM plus LM repair during ACLR at 2 years of follow-up. This study also shows that patients with failed meniscus repair with concomitant ACLR result significantly inferior scores for all 5 KOOS subscales at the 1-year follow-up [34]. Meniscus repair failure rates after ACLR can be as high as 27 % at 5 years of follow-up, according to Nepple et al. [56] In contrast to this fact Robb et al. found at 2-year follow-up, that patients who had meniscectomy during ACL reconstruction were 4.9 times more likely to have an ACLR failure [57].

We analyzed the KOOS score changes of meniscectomy and meniscus repair separately for the lateral and medial meniscal injuries. We found similar pooled results for both sides. This is in contrast with the report of Cox et al. who found that the lateral meniscus repairs did not correlate with inferior results after ACLR, but medial meniscus repairs predicted worse IKDC and KOOS scores 6 years after surgery [58].

Michalitis et al. did a 2-year MRI follow-up after ACLR and compared the meniscal injured patients (meniscectomy and repair combined) with an intact meniscal cohort. Their results showed a multicompartmental pattern of cartilage involvement, and the lateral compartment was the most severely affected. By their analysis, partial meniscectomy of the medial meniscus appeared to be a risk factor for the progression of chondral lesions inside the knee joint [43].

Casp et al. has reported on bilateral isokinetic and isometric strength tests of the knee extensor and flexor groups after 6 months post-ACLR. Based on their results, weightbearing and range of motion restrictions associated with meniscal repair recovery did not result in loss of early strength or worse patient-reported outcomes compared to meniscectomy [32]. Lepley et al. also presented similar results. At the time of return to sports, they found that concomitant meniscectomy or meniscal repair did not affect the recovery of quadriceps activation following ACLR (Quadriceps activation 88.2 ± 9.1 vs. 85.2 ± 9.2 (mean ± SD)). However, all participants demonstrated levels of quadriceps activation failure that are below that of healthy individuals. Therefore, they recommend developing and employing rehabilitation protocols that target quadriceps activation failure post-reconstruction [41].

Wang et al. performed kinematic assessment at a 2-year follow-up. They found that ACLR with concurrent medial meniscectomy demonstrated larger adduction and external tibial rotation than in the case of meniscal repair during level walking, and suspected that this could be one of the reasons behind increased osteoarthritic changes. However, they did not find any radiographical differences [49].

Lastly, not all meniscal tears need surgical intervention during ACLR. Based on Balazs et al. results, patients with an unstable ramp lesion who underwent repair had a significantly higher risk of reoperation for medial meniscal pathology than patients with an untreated stable ramp lesion (22 % vs. 3 %, *p* = 0.03). However, they also found that patients with untreated stable ramp lesions had similar clinical outcomes to those without a ramp lesion, so it was concluded that treating stable ramp lesions at the time of ACLR did not have clinical benefits [30].

### 4.1 Strengths and limitation

Regarding the strengths of our analysis, we followed our protocol, which was registered in advance, while we applied a rigorous methodology.

On the other hand, our study has several limitations. First, no randomized controlled trials were available for inclusion in our analysis. Second, we included a low number of studies with varied outcomes, although our quantitative analysis covered a considerable number of patients. Third, the follow-up periods differed significantly among the studies, ranging from months to years. Additionally, the included studies did not differentiate subgroups based on the mechanism of injury, the type and localization of meniscal tears, or specific age groups. The studies were conducted in different healthcare facilities with varying numbers of surgeries performed annually, and there was considerable heterogeneity in the study populations. These limitations should be considered when interpreting our results, as they may impact the generalizability and applicability of the findings.

## 5 Conclusion

Based on our results, we did not find a clinically significant difference between meniscus repair and meniscectomy during ACLR, mainly due to the low level of evidence and the lack of randomized trials assessing this research question. Additionally, the follow-up period was short in most cases.

## 6 Implications for practice and research

Previously, the benefit of rapid integration of results into clinical practice has been proved [59,60].

We recommend the use of both meniscus repair and meniscectomy techniques in ACLR, tailored to individual patient characteristics. However, there is a critical need for randomized controlled trials to assess the effectiveness of each intervention and compare them to non-operative management. Additionally, high-quality, long-term follow-up studies are essential to evaluate the impact of these interventions on osteoarthritis development. These studies should consider the types and locations of meniscal injuries as well as the specific surgical techniques employed. Lastly, risk groups that benefit the best from each intervention should be identified.

## Funding

Sponsors had no role in the design, data collection, analysis, interpretation, or manuscript preparation.

## Ethical approval

No ethical approval was required for this systematic review with meta-analysis, as all data were already published in peer-reviewed journals. No patients were involved in the design, conduct, or interpretation of our study.

The datasets used in this study can be found in the full-text articles included in the systematic review and meta-analysis.

## CRediT authorship contribution statement

**Gyula Ferenc Szőcs:** Conceptualization, Formal analysis, Writing – original draft. **Szilárd Váncsa:** Conceptualization, Project administration, Methodology, Writing – original draft. **Gergely Agócs:** Conceptualization, Formal analysis, Visualization, Writing – review & editing. **Péter Hegyi:** Conceptualization, Funding acquisition, Writing – review & editing. **Dóra Matis:** Conceptualization, Data curation, Writing – review & editing. **Gergely Pánics:** Conceptualization, Data curation, Writing – review & editing. **Zoltán Bejek:** Conceptualization, Writing – review & editing. **György Márk Hangody:** Conceptualization, Supervision, Writing – original draft.

## Declaration of competing interest

A conflict of interest occurs when an individual's objectivity is potentially compromised by a desire for financial gain, prominence, professional advancement or a successful outcome. The Editors of the Journal of Orthopaedic Translation strive to ensure that what is published in the Journal is as balanced, objective and evidence-based as possible. Since it can be difficult to distinguish between an actual conflict of interest and a perceived conflict of interest, the Journal requires authors to disclose all and any potential conflicts of interest.
