# Supplementary material for: Does concomitant meniscus repair and meniscectomy show different efficacy in anterior cruciate ligament reconstruction? A systematic review and meta-analysis
Source: J Orthop Translat. 2024 Jul 25;48:1–10. doi: 10.1016/j.jot.2024.07.004 (PMC11327393; doi:10.1016/j.jot.2024.07.004)
Supplement: Multimedia component 1 [file mmc1.docx]

**Supplementary Materials**

**Title**

Does concomitant meniscus repair and meniscectomy show different efficacy in anterior cruciate ligament reconstruction? A systematic review and meta-analysis

**Authors**

Gyula Ferenc Szőcs^1,2^, Szilárd Váncsa^2,3,4^, Gergely Agócs^2,5^, Péter Hegyi^2,3,4^, Dóra Matis^2^, Gergely Pánics^1^, Zoltán Bejek^6^, György Márk Hangody^1,2^

**Affiliations:**

1. Department of Orthopaedic Surgery and Traumatology, Uzsoki Hospital, Budapest, Hungary
2. Centre for Translational Medicine, Semmelweis University, Budapest, Hungary
3. Institute for Translational Medicine, Medical School, University of Pécs, Pécs, Hungary
4. Institute of Pancreatic Diseases, Semmelweis University, Budapest, Hungary
5. Department of Biophysics and Radiation Biology, Semmelweis University, Budapest, Hungary
6. Department of Orthopaedics, Semmelweis University, Budapest, Hungary

**TABLE OF CONTENT I.**

**Supplementary Figure 1.** Forest plots representing the after intervention results in the different KOOS subscales between meniscus repair and meniscectomy in addition to primary ACLR

**Supplementary Figure 2.** Forest plots representing the after intervention results in the different KOOS subscales between meniscus repair and meniscectomy for (A) medial and (B) lateral meniscus injuries in addition to primary ACLR

**Supplementary Figure 3.** Forest plots representing the after intervention results in (A) IKDC and (B) Lysholm score between meniscus repair and meniscectomy in addition to primary ACLR

**Supplementary Figure 4.** Forest plots representing the (A) mean changes and (B) after-intervention results for anterior tibial translation measured by arthrometry between meniscus repair and meniscectomy in addition to primary ACLR

**Supplementary Table 1**. PRISMA checklist

**Supplementary Table 2**. Eligibility criteria for each included study

**Supplementary Table 3**. Risk of bias assessment using the ROBINS-I tool

**Supplementary Table 4-6**. Quality of evidence for the assessed outcomes

**
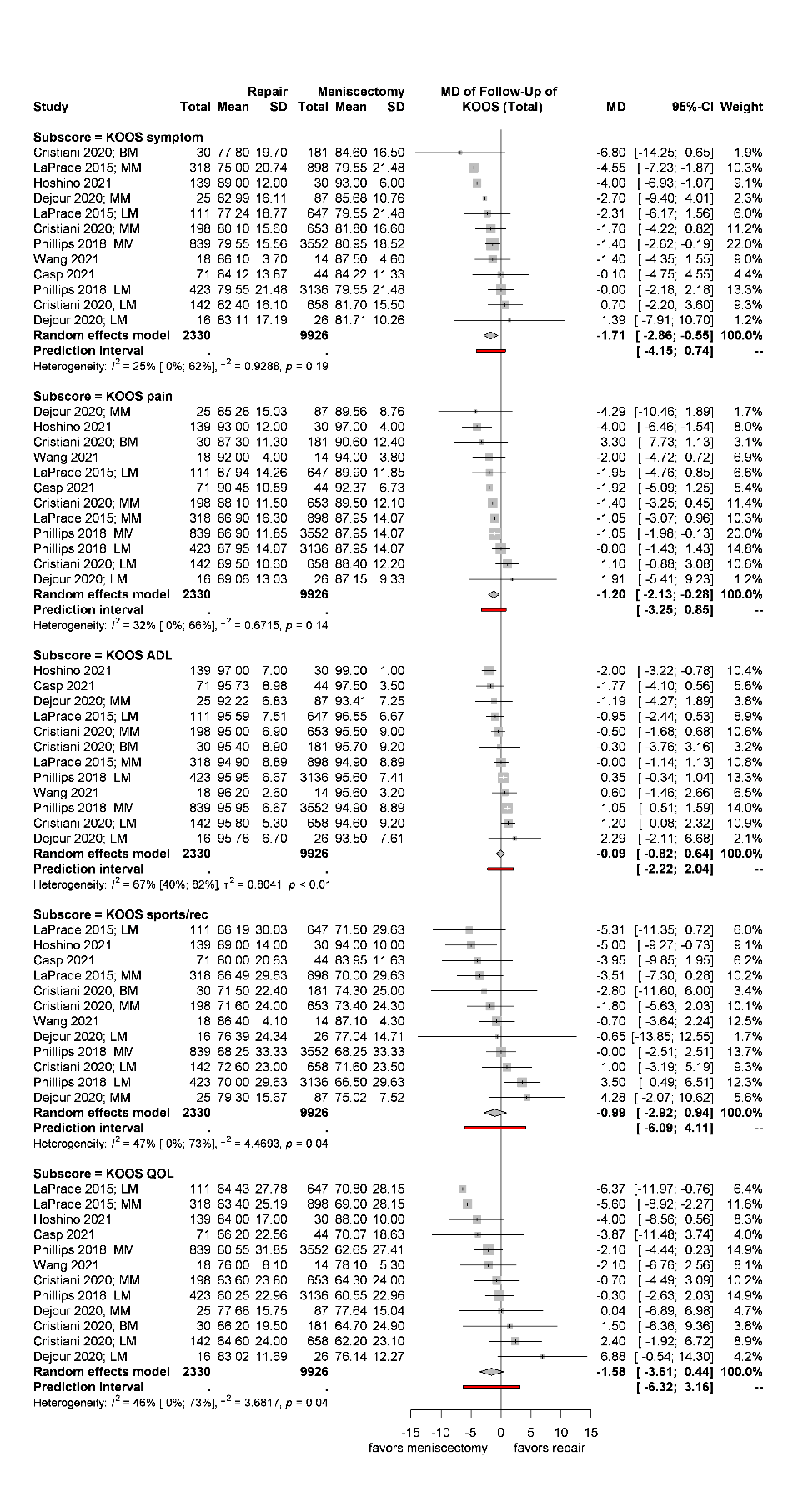
**

**Supplementary Figure 1.** Forest plots representing the after intervention results in the different KOOS subscales between meniscus repair and meniscectomy in addition to primary ACLR

**
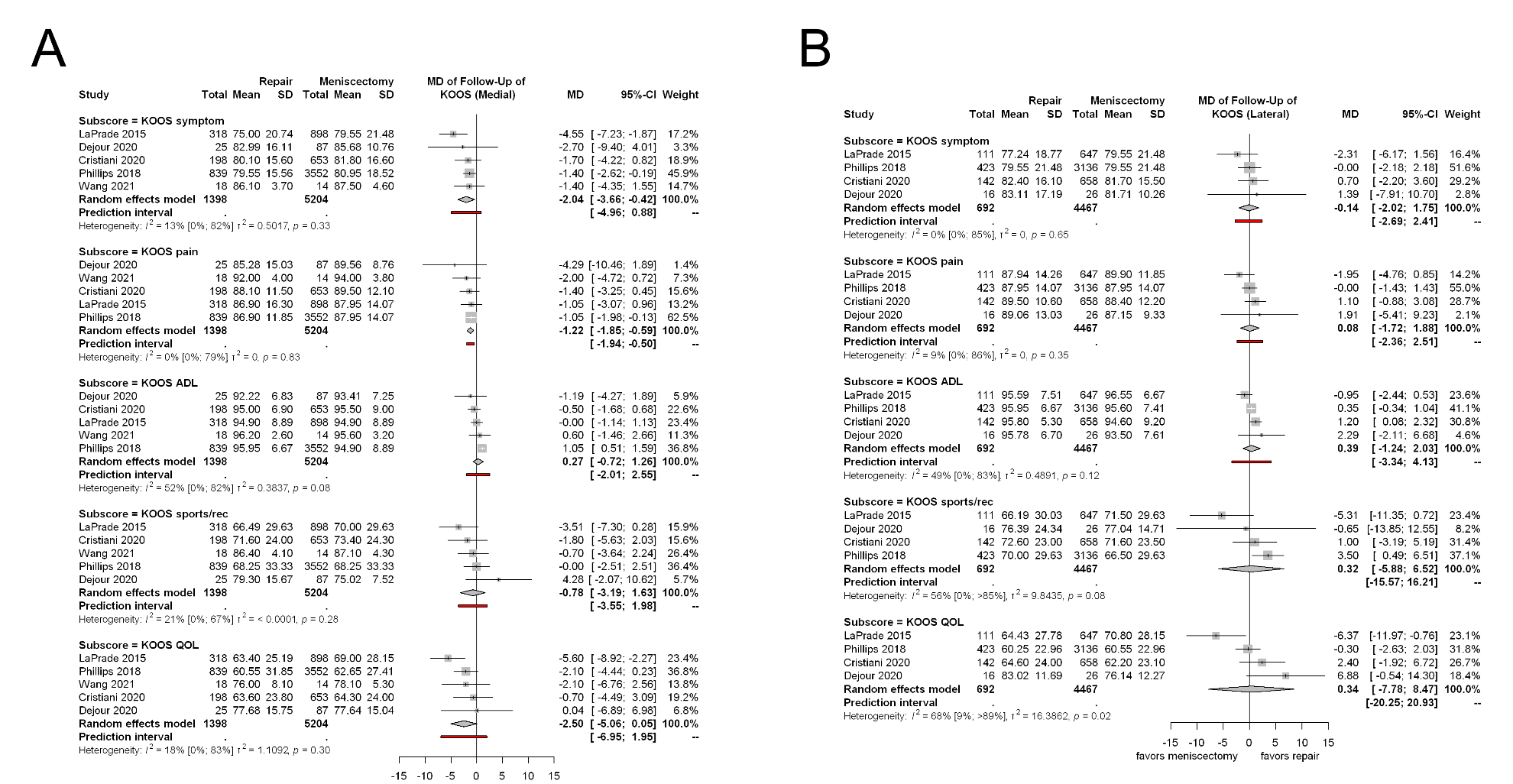
**

**Supplementary Figure 2.** Forest plots representing the after intervention results in the different KOOS subscales between meniscus repair and meniscectomy for (A) medial and (B) lateral meniscus injuries in addition to primary ACLR

**
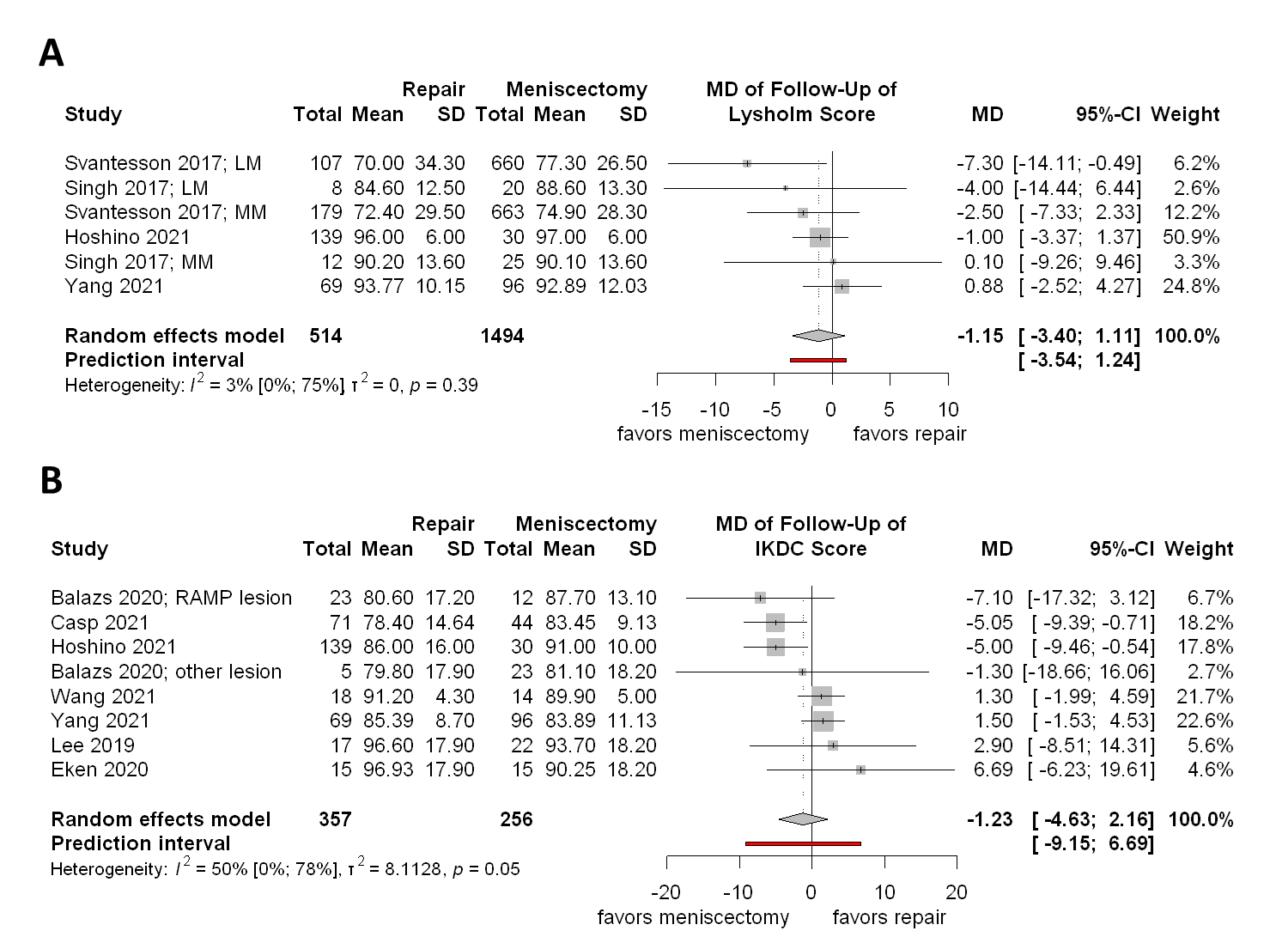
Supplementary Figure 3.** Forest plots representing the after intervention results in (A) IKDC and (B) Lysholm score between meniscus repair and meniscectomy in addition to primary ACLR

**
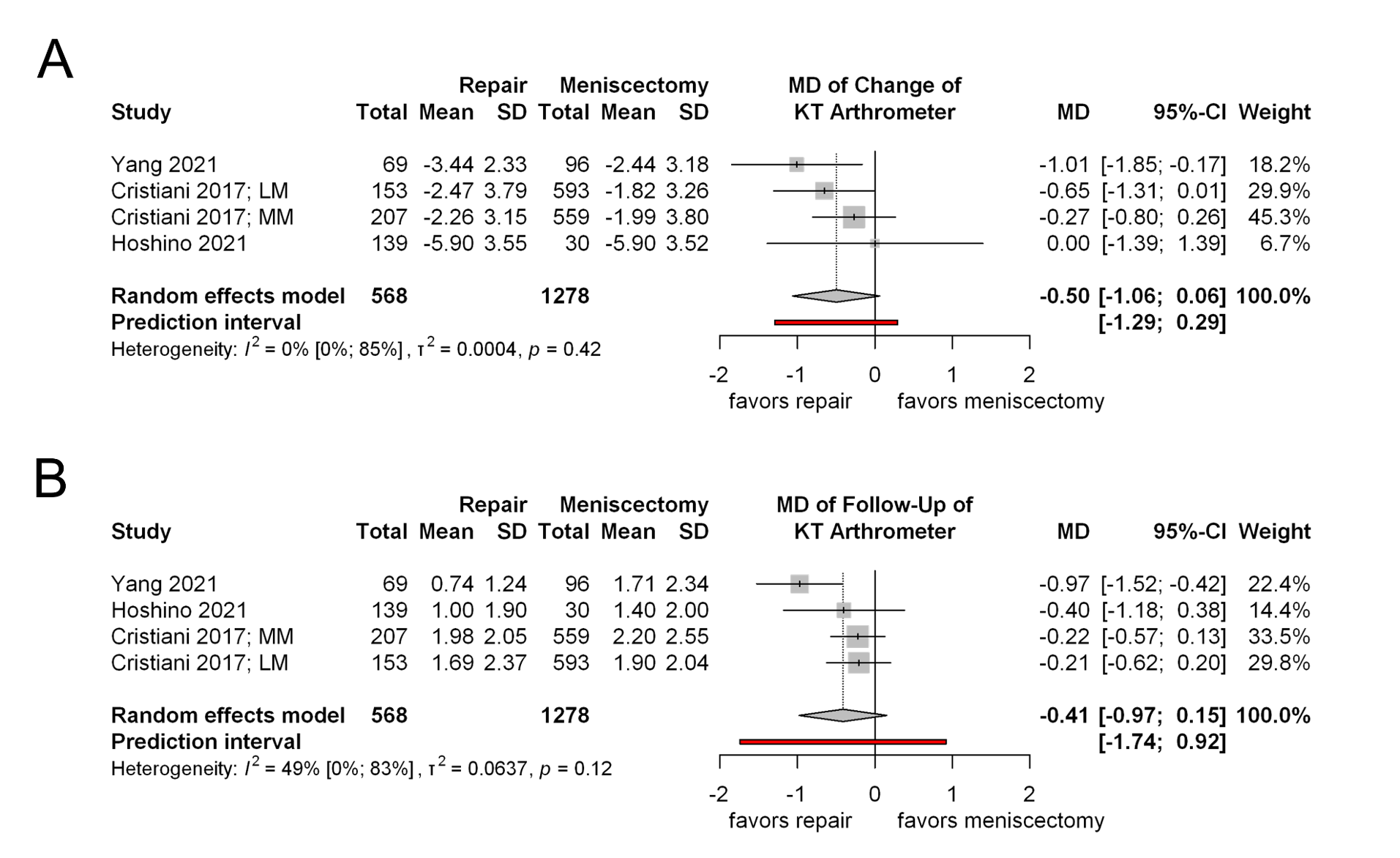
**

**Supplementary Figure 4.** Forest plots representing the (A) mean changes and (B) after-intervention results for anterior tibial translation measured by arthrometry between meniscus repair and meniscectomy in addition to primary ACLR

**Supplementary Table 1.** PRISMA 2020 checklist

| **Section and topic** | **Item #** | **Checklist item** | **Location where item is reported** |  |
| --- | --- | --- | --- | --- |
| **Title** | | | | |
| Title | 1 | Identify the report as a systematic review. | 1 |  |
| **Abstract** | | | | |
| Abstract | 2 | See the PRISMA 2020 for Abstracts checklist (table 2). | 6 |  |
| **Introduction** | | | | |
| Rationale | 3 | Describe the rationale for the review in the context of existing knowledge. | 8 |  |
| Objectives | 4 | Provide an explicit statement of the objective(s) or question(s) the review addresses. | 8 |  |
| **Methods** | | | | |
| Eligibility criteria | 5 | Specify the inclusion and exclusion criteria for the review and how studies were grouped for the syntheses. | 10 |  |
| Information sources | 6 | Specify all databases, registers, websites, organisations, reference lists and other sources searched or consulted to identify studies. Specify the date when each source was last searched or consulted. | 10 |  |
| Search strategy | 7 | Present the full search strategies for all databases, registers and websites, including any filters and limits used. | 11 |  |
| Selection process | 8 | Specify the methods used to decide whether a study met the inclusion criteria of the review, including how many reviewers screened each record and each report retrieved, whether they worked independently, and if applicable, details of automation tools used in the process. | 11 |  |
| Data collection process | 9 | Specify the methods used to collect data from reports, including how many reviewers collected data from each report, whether they worked independently, any processes for obtaining or confirming data from study investigators, and if applicable, details of automation tools used in the process. | 11 |  |
| Data items | 10a | List and define all outcomes for which data were sought. Specify whether all results that were compatible with each outcome domain in each study were sought (e.g. for all measures, time points, analyses), and if not, the methods used to decide which results to collect. | 10 |  |
|  | 10b | List and define all other variables for which data were sought (e.g. participant and intervention characteristics, funding sources). Describe any assumptions made about any missing or unclear information. | 12 |  |
| Study risk of bias assessment | 11 | Specify the methods used to assess risk of bias in the included studies, including details of the tool(s) used, how many reviewers assessed each study and whether they worked independently, and if applicable, details of automation tools used in the process. | 12 |  |
| Effect measures | 12 | Specify for each outcome the effect measure(s) (e.g. risk ratio, mean difference) used in the synthesis or presentation of results. | 12 |  |
| Synthesis methods | 13a | Describe the processes used to decide which studies were eligible for each synthesis (e.g. tabulating the study intervention characteristics and comparing against the planned groups for each synthesis (item #5)). | 12 |  |
|  | 13b | Describe any methods required to prepare the data for presentation or synthesis, such as handling of missing summary statistics, or data conversions. | 12 |  |
|  | 13c | Describe any methods used to tabulate or visually display results of individual studies and syntheses. | 12 |  |
|  | 13d | Describe any methods used to synthesise results and provide a rationale for the choice(s). If meta-analysis was performed, describe the model(s), method(s) to identify the presence and extent of statistical heterogeneity, and software package(s) used. | 12 |  |
|  | 13e | Describe any methods used to explore possible causes of heterogeneity among study results (e.g. subgroup analysis, meta-regression). | 12 |  |
|  | 13f | Describe any sensitivity analyses conducted to assess robustness of the synthesised results. | 12 |  |
| Reporting bias assessment | 14 | Describe any methods used to assess risk of bias due to missing results in a synthesis (arising from reporting biases). | 12 |  |
| Certainty assessment | 15 | Describe any methods used to assess certainty (or confidence) in the body of evidence for an outcome. | 13 |  |
| **Results** | | | | |
| Study selection | 16a | Describe the results of the search and selection process, from the number of records identified in the search to the number of studies included in the review, ideally using a flow diagram (see fig 1). | 14 |  |
|  | 16b | Cite studies that might appear to meet the inclusion criteria, but which were excluded, and explain why they were excluded. | 14 |  |
| Study characteristics | 17 | Cite each included study and present its characteristics. | 14 |  |
| Risk of bias in studies | 18 | Present assessments of risk of bias for each included study. | Suppl. |  |
| Results of individual studies | 19 | For all outcomes, present, for each study: (a) summary statistics for each group (where appropriate) and (b) an effect estimate and its precision (e.g. confidence/credible interval), ideally using structured tables or plots. | Figures |  |
| Results of syntheses | 20a | For each synthesis, briefly summarise the characteristics and risk of bias among contributing studies. | 14-16 |  |
|  | 20b | Present results of all statistical syntheses conducted. If meta-analysis was done, present for each the summary estimate and its precision (e.g. confidence/credible interval) and measures of statistical heterogeneity. If comparing groups, describe the direction of the effect. | 14-16 |  |
|  | 20c | Present results of all investigations of possible causes of heterogeneity among study results. | 14-16 |  |
|  | 20d | Present results of all sensitivity analyses conducted to assess the robustness of the synthesised results. | 14-16 |  |
| Reporting biases | 21 | Present assessments of risk of bias due to missing results (arising from reporting biases) for each synthesis assessed. | 14-16 |  |
| Certainty of evidence | 22 | Present assessments of certainty (or confidence) in the body of evidence for each outcome assessed. | 14-16 |  |
| **Discussion** | | | | |
| Discussion | 23a | Provide a general interpretation of the results in the context of other evidence. | 18 |  |
|  | 23b | Discuss any limitations of the evidence included in the review. | 19-20 |  |
|  | 23c | Discuss any limitations of the review processes used. | 19-20 |  |
|  | 23d | Discuss implications of the results for practice, policy, and future research. | 19-20 |  |
| **Other information** | | | | |
| Registration and protocol | 24a | Provide registration information for the review, including register name and registration number, or state that the review was not registered. | 10 |  |
|  | 24b | Indicate where the review protocol can be accessed, or state that a protocol was not prepared. | 10 |  |
|  | 24c | Describe and explain any amendments to information provided at registration or in the protocol. | 10 |  |
| Support | 25 | Describe sources of financial or non-financial support for the review, and the role of the funders or sponsors in the review. | 3 |  |
| Competing interests | 26 | Declare any competing interests of review authors. | 3 |  |
| Availability of data, code, and other materials | 27 | Report which of the following are publicly available and where they can be found: template data collection forms; data extracted from included studies; data used for all analyses; analytic code; any other materials used in the review. | 3 |  |

**Supplementary Table 2**. Eligibility criteria for each included study

| **Author (year)** | **Inclusion** | **Exclusion** |
| --- | --- | --- |
| Balazs et al. (2020) [1] | patients who underwent surgical treatment of an ACL rupture over the specified time period and had a preoperative MRI performed at our facility available for review. + at least 1-year follow-up | "Patients were excluded if they had less than 1-year clinical follow-up, had underlying conditions that would increase their risk for failure of ACL reconstruction or meniscal healing (e.g., any history of ipsilateral knee surgery or diabetes), sustained a multi-ligamentous knee injury, underwent a double-bundle ACL reconstruction, did not undergo ACL reconstruction at the time of index surgery, or did not complete baseline patient-reported outcome questionnaires. |
| Byrne et al. (2021) [2] | Participants included were male, multidirectional athletes between the ages of 16–35 years undergoing primary ACL reconstruction with a bone-patella tendon-bone autograft. Multi-directional sports require frequent and consistent sagittal plane movement and running as well as lateral shuffling, cutting and jumping [11]. The primary sports included in this study were Gaelic football (52%), soccer (18%), hurling (16%) and rugby (14%). Participants were only eligible for to participate in this study if they underwent testing 9–11 months after ACLR as part of a standardised return to play assessment protocol. | Exclusion criteria were revision ACL surgery and multi ligament reconstruction. Participants undergoing partial meniscectomy or repair of both compartments were included and were placed in the appropriate meniscectomy and repair groups. Those who underwent a meniscectomy in one compartment of the knee but also underwent meniscal repair in the other compartment (n = 2) were also excluded from the study. |
| Casp et al. (2021) [3] | "We included patients who had a primary ACLR with no surgical complications and no history of prior ACLR or contralateral ACL injury." + Chondral fraying requiring only mechanical shaving chondroplasty was not excluded from the analysis." | Patients with concomitant surgical ligamentous injuries or full-thickness chondral injuries were excluded as confounding" |
| Cristiani et al. (2017) [4] | Inclusion criteria for this study were years of surgery from 2000 to 2015, no concomitant posterior cruciate ligament ro posterolateral corner injuries, and no surgically treated medial collateral ligament injuries. | Exclusion criteria were meniscus treatments (n=88), such as meniscus repair during a resection of the contralateral meniscus, as well as repair of both menisci (n=34) and contralateral knee ligament surgery (n=359). Moreover, patients with no pre- or postoperative KT-1000 arthrometer (MEDmetric Corp) values available were excluded (n=919). |
| Cristiani et al. (2020) [5] | "patients who underwent primary ACLR, with no concomitant ligament injuries, at our clinic from January 2005 to December 2015 were assessed for eligibility" | "Patients fulfilling any of the following criteria were excluded: ACL graft rupture or revision ACLR; contralateral ACL injury or reconstruction; meniscus injury without treatment; and combinations of the meniscus treatments, such as meniscus repair during resection of the contralateral meniscus." |
| Dejour et al. (2020) [6] | "The inclusion criteria were (i) primary ACLR in (ii) patients aged over 50 years. " | "The exclusion criteria were (iii) severe concomitant ligament injury (grade 3), (iv) tibiofemoral osteoarthritis visible on weight-bearing anteroposterior and/or sagittal radiographs (>50% joint space narrowing), and (v) history of intra- or extra-articular ligament surgery in either knee. " |
| Eken et al. (2020) [7] | " Inclusion criteria were: (1) patients with concomitant ACL rupture and neglected or delayed medial meniscus tear, (2) outerbridge grade 2 or lower grade chondral injury, (3) in addition to ACL rupture, having medial meniscal bucket-handle tear for more than 6 months, and (4) patients aged between 18 and 45 years. | "Of the 42 patients, 12 patients with previous meniscal or ACL surgery, contralateral ACL injury, tears within white-white zone, and concurrent treatment for osteochondral or chondral lesions were excluded." |
| Hatayama et al. (2020) [8] | "patients who underwent primary anatomic double-bundle ACL reconstruction by use of the semitendinosus tendon at our institute were included in our study. Patients who had a grade 1 or 2 medial collateral ligament injury were not excluded" | "Patients were excluded from our study if they had single-bundle reconstruction, revision ACL reconstruction, bilateral ACL injuries, or multiple ligamentous injuries." |
| Hoshino et al. (2021) [9] | patients who underwent primary ACL reconstruction using autologous hamstring graft between August 2013 and October 2016 registered in our study database and who were followed up for 2 years were included. | exclusion criteria comprised [1] prior injuries or surgeries in the contralateral knee [2], prior ligamentous injuries in the involved knee [3], grade 2 or 3 concomitant ligament injuries, and [4] inflammatory or other forms of osteoarthritis. Data from the preoperative period and at 2-year follow up were used for further analysis, and patients with incomplete data, patients who were lost to follow-up, and patients who had re-injury within 2 years after surgery were also excluded |
| Kacmaz et al. (2021) [10] | Adult patients operated by the same surgeon using the same surgical technique with at least 1 year of follow-up, who participated in subjective knee evaluations  during outpatient follow-up and with pathology limited to one knee were included in the study | Patients with accompanying lesions other than meniscus and anterior cruciate ligament injury (osteochondral lesion, multiple ligament lesion), revision surgery, bone patellar tendon bone ACL reconstruction, inside-out and outside-in meniscus repair and were in the pediatric age group were excluded |
| LaPrade et al. (2015) [11] | A total of 14,142 patients with primary ACLR from 2004 to 2012 were available in the NKLR database | patients with concomitant medial collateral ligament (MCL), posterior cruciate ligament (PCL), posterolateral corner injuries, or meniscal transplantations (n = 1198) were excluded. Patients who had surgeries performed in 2011 or 2012 and lacked a 2-year follow-up were also excluded (n = 3185). Patients with any concomitant meniscal procedures performed (n = 561), such as a resection of the contralateral compartment meniscus during a meniscal repair, were excluded in these groups, as well as patients with untreated meniscal injury (n = 594). Patients with untreated meniscal injury were excluded from further analysis because of a lack of standardization toward reporting which meniscus was injured and left untreated. In addition, patients for whom no preoperative KOOS data were collected were excluded (n = 1191). |
| Lee et al. (2019) [12] | Potential patients at a single large tertiary hospital in Singapore who underwent either partial meniscectomy or meniscal repair (with or without ACL injury and reconstruction) between 2008 and 2016 by a single surgeon were considered for inclusion in the study | The following patient groups were excluded: (1) previous meniscal surgery done prior to current meniscal surgery, (2) concomitant ACL injury without undergoing simultaneous ACL reconstruction (patients with ACLdeficient knees), (3) posterior cruciate ligament injury and (4) underwent meniscal transplant. |
| Lepley et al. (2014) [13] | Forty-six individuals that have been cleared for participation following ACL reconstruction were invited to participate in this retrospective study | Potential participants were excluded if they: had a previous history of surgery (other than the ACL reconstruction) to either knee, suffered a previous ACL injury, or had a known heart condition. Pregnant females were also excluded. |
| Melton et al. (2011) [14] | 44 patients (Group R) who had undergone simultaneous meniscal repair and ACL reconstruction between 1991 and 1999 were identified | Any patients who had undergone simultaneous treatment for chondral or osteochondral lesions were not included in the patient cohort. Of the 44 patients, 9 were excluded for previous meniscal or ACL surgery |
| Michalitsis et al. (2016) [15] | Twenty-nine patients (mean age 30.3 ± 10 years) of whom 26 were male (mean age 30.8 ± 9.8) and 3 were female (mean age 26.7 ± 13.4 years) were enrolled in the present study. | four patients were excluded due to ACL graft failure and another one due to post-operative infection that resulted in early revision arthroscopy |
| Philips et al. (2018) [16] | (1) An index ACL reconstruction from 1st January 2005 to 31^st^ December 2014, (2) Age older than 12 years, (3) Isolated primary ACL reconstruction with autograft, (4) No ACL revision or contralateral ACL injury, (5) Non-treated medial collateral ligament (MCL) or lateral collateral ligament (LCL) injury, (6) No associated injury (fracture, nerve or vascular injury, tendon rupture), (7) Patients with KOOS and EQ-5D subscales available at 2 years following ACLR, and (8) No additional meniscus surgery post-ACL reconstruction on ipsilateral or contralateral side. IRB approval was obtained from the Stockholm Regional Ethical Review Board for the data collection within the database (IRB number: 2011/337-31/3). | There were 7670 (33.26%) patients excluded from this analysis due to not meeting the pre-defined inclusion criteria |
| Shelbourne (2003) [17] | Between 1982 and 1995, the senior author (KDS) performed 2096 ACL reconstructions with use of patellar tendon autografts. We conducted a retrospective review of the prospectively collected data. One hundred fifty-five patients met the inclusion criteria of ACL deficiency with an isolated unstable bucket-handle medial meniscal tear involving 50% or more of the circumference of the meniscus. | Patients were excluded if they had associated articular cartilage damage of greater than Outerbridge14 grade 2 in any compartment, a lateral meniscal tear or degeneration, or additional ligament injury. |
| Shelbourne (2004) [18] | Between 1982 and 1995, 2,065 patients underwent ACL reconstruction. From a prospective database of these patients, we identified 472 patients who had a bucket-handle lateral meniscus tear. | patients who had either grade 3 or 4 chondromalacia in any compartment or other meniscal lesions |
| Singh et al. (2017) [19] | Between January 2009 and December 2012, consecutive patients undergoing primary ACL reconstruction with quadrupled hamstring autograft at our institution were recruited. | Patients with multi-ligament knee injuries, concomitant knee injuries (apart from meniscal injury), revision ACLR or any previous knee surgeries were excluded from this study. |
| Svantesson et al. (2017) [20] | Patients who underwent primary ACL reconstruction at Capio Artro Clinic, Stockholm, Sweden, between 1^st^ Jan 2001 and 31st Dec 2014 without concomitant injuries others than meniscal and/or cartilage lesions were included. | Patients with contralateral or revision ACL reconstruction were excluded, as well as patients with untreated meniscal injury and patients receiving more than one type of meniscal treatment such as combinations of repair and resection. |
| Wang et al. (2021) [21] | Between January 2014 and December 2016, 161 patients requiring primary ACL reconstruction with hamstring tendon autograft who also had medial meniscal tears underwent surgery at our institution. Patients were recruited using the following inclusion criteria: (1) primary ACL reconstruction with hamstring autograft, (2) medial meniscal tears treated with meniscal repair or partial meniscectomy, (3) willingness to return to the hospital for kinematic assessment, and (4) age older than 18 years. | Exclusion criteria were (1) simultaneous lateral meniscal tears (n = 45), (2) previous surgery to contralateral limb (n = 5), (3) multiligament injuries (n = 31), (4) worse than grade 2 chondral lesion (n = 11), (5) a combination of meniscal treatments (meniscal repair and partial meniscectomy; n = 16), (6) second knee injury after ACL surgery (n = 3), (7) open physes (n = 3), and (8) body mass index .25 (n = 8) |
| Yang et al. (2021) [22] | all patients who underwent meniscus surgery and simultaneous ACL reconstruction between May 2013 and July 2018. The inclusion criteria were as follows: (1) patients with intraoperatively confirmed ACL rupture combined with medial, lateral, or medial and lateral meniscal injury; (2) aged <60 years; and (3) no history of previous ipsilateral knee meniscal injury. | (1) ACL rupture associated with fracture, collateral ligament injury, or complex ligament injury; (2) a history of knee surgery; or (3) a significant degree of osteoarthritis. |

**Supplementary Table 3**. Risk of bias assessment using the ROBINS-I tool

| **Author** | **OUTCOME** | **D1** | **D2** | **D3** | **D4** | **D5** | **D6** | **D7** | **Overall** |
| --- | --- | --- | --- | --- | --- | --- | --- | --- | --- |
| Balazs et al. | PRO score | low | moderate | low | low | low | serious | moderate | **serious** |
|  | SF12 PCS | low | moderate | low | low | low | serious | moderate | **serious** |
|  | SF12 MCS | low | moderate | low | low | low | serious | moderate | **serious** |
|  | IKDC | low | moderate | low | low | low | serious | moderate | **serious** |
|  | Marx activity scale | low | moderate | low | low | low | serious | moderate | **serious** |
| Byrne et al. | IKDC | low | moderate | low | low | low | serious | moderate | **serious** |
|  | Marx score | low | moderate | low | low | low | serious | moderate | **serious** |
|  | ACL RSI | low | moderate | low | low | low | serious | moderate | **serious** |
|  | Strength and jump performance metrics | low | moderate | low | low | low | moderate | moderate | **moderate** |
|  | Return to play | low | moderate | low | low | low | moderate | moderate | **moderate** |
| Casp et al. | KOOS | low | moderate | moderate | low | low | serious | moderate | **serious** |
|  | IKDC | low | moderate | moderate | low | low | serious | moderate | **serious** |
|  | Knee extension peak torque N*m/kg | low | moderate | moderate | low | low | moderate | moderate | **moderate** |
|  | Knee flexion peak torque N*m/kg | low | moderate | moderate | low | low | moderate | moderate | **moderate** |
|  | LSI: extension | low | moderate | moderate | low | low | moderate | moderate | **moderate** |
|  | LSI: flexion | low | moderate | moderate | low | low | moderate | moderate | **moderate** |
|  | Single-leg hop m/m | low | moderate | moderate | low | low | moderate | moderate | **moderate** |
|  | Triple hop m/m | low | moderate | moderate | low | low | moderate | moderate | **moderate** |
|  | 6-m timed hop | low | moderate | moderate | low | low | moderate | moderate | **moderate** |
|  | LSI: single hop | low | moderate | moderate | low | low | moderate | moderate | **moderate** |
|  | LSI: triple hop | low | moderate | moderate | low | low | moderate | moderate | **moderate** |
|  | LSI: 6-m timed hop | low | moderate | moderate | low | low | moderate | moderate | **moderate** |
|  | Tegner | low | moderate | moderate | low | low | serious | moderate | **serious** |
| Cristiani et al. (2017) | KT-1000 | low | moderate | low | low | low | low | moderate | **moderate** |
| Cristiani et al. (2020) | KOOS | low | moderate | low | low | low | serious | moderate | **serious** |
| Dejour et al. | KOOS medial | moderate | moderate | low | low | low | serious | moderate | **serious** |
|  | KOOS lateral | moderate | moderate | low | low | low | serious | moderate | **serious** |
| Eken et al. | Lysholm | low | moderate | low | low | low | serious | moderate | **serious** |
|  | IKDC | low | moderate | low | low | low | serious | moderate | **serious** |
|  | HSS knee score | low | moderate | low | low | low | serious | moderate | **serious** |
|  | Tegner | low | moderate | low | low | low | serious | moderate | **serious** |
|  | Lachman | low | moderate | low | low | low | moderate | moderate | **moderate** |
|  | Pivot shift | low | moderate | low | low | low | moderate | moderate | **moderate** |
|  | Range of motion | low | moderate | low | low | low | moderate | moderate | **moderate** |
|  | McMurray test | low | moderate | low | low | low | moderate | moderate | **moderate** |
| Hatayama et al. | Lysholm | moderate | moderate | low | moderate | low | serious | moderate | **serious** |
|  | Pivotshift | moderate | moderate | low | moderate | low | moderate | moderate | **moderate** |
|  | Tegner | moderate | moderate | low | moderate | low | serious | moderate | **serious** |
|  | SSD of anterior tibial translation | moderate | moderate | low | moderate | low | low | moderate | **moderate** |
| Hoshino et al. | Lysholm | low | moderate | low | low | low | serious | low | **serious** |
|  | IKDC | low | moderate | low | low | low | serious | low | **serious** |
|  | KOOS | low | moderate | low | low | low | serious | low | **serious** |
|  | Lachman | low | moderate | low | low | low | moderate | low | **moderate** |
|  | KT-1000 (mm) | low | moderate | low | low | low | low | low | **moderate** |
|  | Pivot shift | low | moderate | low | low | low | moderate | low | **moderate** |
|  | Medial JSW | low | moderate | low | low | low | moderate | low | **moderate** |
|  | Lateral JSW | low | moderate | low | low | low | moderate | low | **moderate** |
|  | Medial meniscus medial JSW | low | moderate | low | low | low | moderate | low | **moderate** |
|  | Lateral meniscus lateral JSW | low | moderate | low | low | low | moderate | low | **moderate** |
| Kacmaz et al. | Lysholm | moderate | moderate | low | low | moderate | serious | moderate | **serious** |
|  | Tegner | moderate | moderate | low | low | moderate | serious | moderate | **serious** |
|  | KOOS | moderate | moderate | low | low | moderate | serious | moderate | **serious** |
|  | WOMAC | moderate | moderate | low | low | moderate | serious | moderate | **serious** |
|  | FJS-12 | moderate | moderate | low | low | moderate | serious | moderate | **serious** |
| LaPrade et al. | KOOS | low | moderate | low | moderate | moderate | serious | low | **serious** |
| Lee et al. | IKDC | moderate | moderate | low | low | moderate | serious | low | **serious** |
|  | Tegner | moderate | moderate | low | low | moderate | serious | low | **serious** |
| Lepley et al. | Quad activation | moderate | moderate | low | low | low | moderate | low | **moderate** |
|  | Quad isokinetic | moderate | moderate | low | low | low | moderate | low | **moderate** |
|  | Quad isometric | moderate | moderate | low | low | low | moderate | low | **moderate** |
| Melton et al. | IKDC | moderate | moderate | low | low | moderate | serious | low | **serious** |
|  | Lysholm | moderate | moderate | low | low | moderate | serious | low | **serious** |
| Michalitis et al. | Lysholm | moderate | moderate | low | low | moderate | serious | low | **serious** |
|  | Tegner | moderate | moderate | low | low | moderate | serious | low | **serious** |
|  | KOOS | moderate | moderate | low | low | moderate | serious | low | **serious** |
|  | ICRS classification based on MRI | moderate | moderate | low | low | moderate | moderate | low | **moderate** |
| Phillips et al. | KOOS | low | moderate | low | low | moderate | serious | low | **serious** |
|  | EQ-5D | low | moderate | low | low | moderate | serious | low | **serious** |
| Shelbourne et al. (2003) | Noyes score | moderate | moderate | low | low | moderate | serious | low | **serious** |
|  | IKDC | moderate | moderate | low | low | moderate | serious | low | **serious** |
|  | radiographic IKDC | moderate | moderate | low | low | moderate | moderate | low | **moderate** |
| Shelbourne et al. (2004) | Noyes score | moderate | moderate | low | low | moderate | serious | low | **serious** |
|  | IKDC | moderate | moderate | low | low | moderate | serious | low | **serious** |
|  | radiographic IKDC | moderate | moderate | low | low | moderate | moderate | low | **moderate** |
| Singh et al. | Lysholm | low | moderate | low | low | moderate | serious | low | **serious** |
|  | Tegner | low | moderate | low | low | moderate | serious | low | **serious** |
| Svantesson et al. | KOOS | low | moderate | low | low | moderate | serious | low | **serious** |
|  | Lysholm | low | moderate | low | low | moderate | serious | low | **serious** |
| Wang et al. | IKDC | low | moderate | low | low | low | serious | low | **serious** |
|  | KOOS | low | moderate | low | low | low | serious | low | **serious** |
|  | kinematics | low | moderate | low | low | low | moderate | low | **moderate** |
|  | knee-degenartion | low | moderate | low | low | low | moderate | low | **moderate** |
| Yang et al. | VAS | low | moderate | low | low | low | serious | low | **serious** |
|  | Lysholm | low | moderate | low | low | low | serious | low | **serious** |
|  | IKDC | low | moderate | low | low | low | serious | low | **serious** |
|  | Tegner | low | moderate | low | low | low | serious | low | **serious** |
|  | KT-2000 (mm) | low | moderate | low | low | low | moderate | low | **moderate** |

D1: Bias due to confounding , D2: Bias in selection of participants into the study, D3: Bias in classification of interventions, D4: Bias due to deviations from intended interventions, D5: Bias due to missing data, D6: Bias in measurement of outcomes, D7: Bias in selection of the reported result

**Supplementary Table 4.** ACLR and meniscus repair compared to ACLR and meniscectomy for concomitant ACL and meniscus injury

| **Certainty assessment** | | | | | | | **№ of patients** | | **Effect** | | **Certainty** | **Importance** |
| --- | --- | --- | --- | --- | --- | --- | --- | --- | --- | --- | --- | --- |
| **№ of studies** | **Study design** | **Risk of bias** | **Inconsistency** | **Indirectness** | **Imprecision** | **Other considerations** | **ACLR and meniscus repair** | **ACLR and meniscectomy** | **Relative (95% CI)** | **Absolute (95% CI)** |  |  |
| **KOOS symptom (follow-up: range 6 months to 43 months; Scale from: 0 to 100)** | | | | | | | | | | | | |
| 5 | observational studies | serious^a^ | not serious | not serious | not serious | publication bias strongly suspected^b^ | 2218 | 9769 | - | MD **1.16 lower** (2.48 lower to 0.16 higher) | ⨁⨁◯◯ Low |  |
| **KOOS pain (follow-up: range 6 months to 43 months; Scale from: 0 to 100)** | | | | | | | | | | | | |
| 5 | observational studies | serious^a^ | not serious | not serious | not serious | publication bias strongly suspected^b^ | 2218 | 9769 | - | MD **1.6 lower** (2.48 lower to 0.72 lower) | ⨁⨁◯◯ Low |  |
| **KOOS function in daily living (ADL) (follow-up: range 6 to 43; Scale from: 0 to 100)** | | | | | | | | | | | | |
| 5 | observational studies | serious^a^ | not serious | not serious | not serious | publication bias strongly suspected^b^ | 2218 | 9769 | - | MD **0.08 higher** (0.91 lower to 1.07 higher) | ⨁⨁◯◯ Low |  |
| **KOOS function in sport and recreation (Sport/Rec) (follow-up: range 6 to 43; Scale from: 0 to 100)** | | | | | | | | | | | | |
| 5 | observational studies | serious^a^ | not serious | not serious | not serious | publication bias strongly suspected^b^ | 2218 | 9769 | - | MD **1.25 lower** (4.79 lower to 2.3 higher) | ⨁⨁◯◯ Low |  |
| **KOOS knee related Quality of life (QOL) (follow-up: range 6 to 43; Scale from: 0 to 100)** | | | | | | | | | | | | |
| 5 | observational studies | serious^a^ | not serious | not serious | not serious | publication bias strongly suspected^b^ | 2218 | 9769 | - | MD **2.68 lower** (6.13 lower to 0.77 higher) | ⨁⨁◯◯ Low |  |
| **Lysholm Score (follow-up: range 6 months to 43 months; Scale from: 0 to 100)** | | | | | | | | | | | | |
| 4 | observational studies | serious^a^ | not serious | not serious | not serious | publication bias strongly suspected^b^ | 514 | 1494 | - | MD **2.61 lower** (5.51 lower to 0.29 higher) | ⨁⨁◯◯ Low |  |
| **IKDC Score (follow-up: range 6 months to 43 months; Scale from: 0 to 100)** | | | | | | | | | | | | |
| 4 | observational studies | serious^a^ | not serious | not serious | not serious | publication bias strongly suspected^b^ | 254 | 175 | - | MD **1.08 higher** (4.05 lower to 6.21 higher) | ⨁⨁◯◯ Low |  |
| **KT arthrometry (follow-up: range 6 to 43; Scale from: 0 to >5)** | | | | | | | | | | | | |
| 3 | observational studies | serious^a^ | not serious | not serious | not serious | publication bias strongly suspected^b^ | 568 | 1278 | - | MD **0.5 lower** (1.06 lower to 0.06 higher) | ⨁⨁◯◯ Low |  |

**Supplementary Table 5.** ACLR and meniscus repair compared to ACLR and meniscectomy for concomitant ACL and medial meniscus injury

| **Certainty assessment** | | | | | | | **№ of patients** | | **Effect** | | **Certainty** | **Importance** |
| --- | --- | --- | --- | --- | --- | --- | --- | --- | --- | --- | --- | --- |
| **№ of studies** | **Study design** | **Risk of bias** | **Inconsistency** | **Indirectness** | **Imprecision** | **Other considerations** | **ACLR and meniscus repair** | **ACLR and meniscectomy** | **Relative (95% CI)** | **Absolute (95% CI)** |  |  |
| **KOOS symptom (follow-up: range 6 to 43; Scale from: 0 to 100)** | | | | | | | | | | | | |
| 4 | observational studies | serious^a^ | not serious | not serious | not serious | publication bias strongly suspected^b^ | 1373 | 5117 | - | MD **1.76 lower** (3.26 lower to 0.25 lower) | ⨁⨁◯◯ Low |  |
| **KOOS pain (follow-up: range 6 to 43; Scale from: 0 to 100)** | | | | | | | | | | | | |
| 4 | observational studies | serious^a^ | not serious | not serious | not serious | publication bias strongly suspected^b^ | 1373 | 5117 | - | MD **2.01 lower** (3.62 lower to 0.4 lower) | ⨁⨁◯◯ Low |  |
| **KOOS function in daily living (ADL) (follow-up: range 6 to 43; Scale from: 0 to 100)** | | | | | | | | | | | | |
| 4 | observational studies | serious^a^ | not serious | not serious | not serious | publication bias strongly suspected^b^ | 1373 | 5117 | - | MD **0.01 lower** (2.45 lower to 2.42 higher) | ⨁⨁◯◯ Low |  |
| **KOOS function in sport and recreation (Sport/Rec) (follow-up: range 6 to 43; Scale from: 0 to 100)** | | | | | | | | | | | | |
| 4 | observational studies | serious^a^ | not serious | not serious | not serious | publication bias strongly suspected^b^ | 1373 | 5117 | - | MD **2.27 lower** (9.53 lower to 5 higher) | ⨁⨁◯◯ Low |  |
| **KOOS knee related Quality of life (QOL) (follow-up: range 6 to 43; Scale from: 0 to 100)** | | | | | | | | | | | | |
| 4 | observational studies | serious^a^ | not serious | not serious | not serious | publication bias strongly suspected^b^ | 1373 | 5117 | - | MD **2.84 lower** (8.86 lower to 3.18 higher) | ⨁⨁◯◯ Low |  |

**Supplementary Table 6.** ACLR and meniscus repair compared to ACLR and meniscectomy for concomitant ACLR and lateral meniscus injury

| **Certainty assessment** | | | | | | | **№ of patients** | | **Effect** | | **Certainty** | **Importance** |
| --- | --- | --- | --- | --- | --- | --- | --- | --- | --- | --- | --- | --- |
| **№ of studies** | **Study design** | **Risk of bias** | **Inconsistency** | **Indirectness** | **Imprecision** | **Other considerations** | **ACLR and meniscus repair** | **ACLR and meniscectomy** | **Relative (95% CI)** | **Absolute (95% CI)** |  |  |
| **KOOS symptom (follow-up: range 6 to 43; Scale from: 0 to 100)** | | | | | | | | | | | | |
| 3 | observational studies | serious^a^ | not serious | not serious | not serious | publication bias strongly suspected^b^ | 676 | 4441 | - | MD **0.85 higher** (3.58 lower to 5.28 higher) | ⨁⨁◯◯ Low |  |
| **KOOS pain (follow-up: range 6 to 43)** | | | | | | | | | | | | |
| 3 | observational studies | serious^a^ | not serious | not serious | not serious | publication bias strongly suspected^b^ | 676 | 4441 | - | MD **0.76 lower** (1.96 lower to 0.44 higher) | ⨁⨁◯◯ Low |  |
| **KOOS function in daily living (ADL) (follow-up: range 6 to 43; Scale from: 0 to 100)** | | | | | | | | | | | | |
| 3 | observational studies | serious^a^ | not serious | not serious | not serious | publication bias strongly suspected^b^ | 676 | 4441 | - | MD **0.11 higher** (2.8 lower to 3.02 higher) | ⨁⨁◯◯ Low |  |
| **KOOS function in sport and recreation (Sport/Rec) (follow-up: range 6 to 43)** | | | | | | | | | | | | |
| 3 | observational studies | serious^a^ | not serious | not serious | not serious | publication bias strongly suspected^b^ | 676 | 4441 | - | MD **0.07 higher** (12.3 lower to 12.43 higher) | ⨁⨁◯◯ Low |  |
| **KOOS knee related Quality of life (QOL) (follow-up: range 6 to 43; Scale from: 0 to 100)** | | | | | | | | | | | | |
| 3 | observational studies | serious^a^ | not serious | not serious | not serious | publication bias strongly suspected^b^ | 676 | 4441 | - | MD **1.12 lower** (8.76 lower to 6.51 higher) | ⨁⨁◯◯ Low |  |

**CI:** confidence interval; **MD:** mean difference

#### Explanations

a. moderate risk of bias

b. retrospective and prospective observational studies

**REFERENCES**

1. Balazs GC, Greditzer HG, Wang D, Marom N, Potter HG, Rodeo SA, et al. Non-treatment of stable ramp lesions does not degrade clinical outcomes in the setting of primary ACL reconstruction. Knee surgery, sports traumatology, arthroscopy : official journal of the ESSKA 2020;28(11):3576-86. [English].

2. Byrne L, King E, Mc Fadden C, Jackson M, Moran R, Daniels K. The effect of meniscal pathology and management with ACL reconstruction on patient-reported outcomes, strength, and jump performance ten months post-surgery. Knee 2021;32:72-79. [English].

3. Casp AJ, Bodkin SG, Gwathmey FW, Werner BC, Miller MD, Diduch DR, et al. Effect of Meniscal Treatment on Functional Outcomes 6 Months After Anterior Cruciate Ligament Reconstruction. Orthop J Sports Med 2021;9(10):23259671211031281. [eng].

4. Cristiani R, Rönnblad E, Engström B, Forssblad M, Stålman A. Medial Meniscus Resection Increases and Medial Meniscus Repair Preserves Anterior Knee Laxity: A Cohort Study of 4497 Patients With Primary Anterior Cruciate Ligament Reconstruction. The American journal of sports medicine 2018;46(2):357-62. [English].

5. Cristiani R, Parling A, Forssblad M, Edman G, Engström B, Stålman A. Meniscus Repair Does Not Result in an Inferior Short-term Outcome Compared With Meniscus Resection: An Analysis of 5,378 Patients With Primary Anterior Cruciate Ligament Reconstruction. Arthroscopy - Journal of Arthroscopic and Related Surgery 2020;36(4):1145-53. [English].

6. Dejour D, de Lavigne C, Panisset JC, Gonzalez JF, Ode Q, Ehlinger M, et al. Female gender and medial meniscal lesions are associated with increased pain and symptoms following anterior cruciate ligament reconstruction in patients aged over 50 years. Knee surgery, sports traumatology, arthroscopy : official journal of the ESSKA 2021;29(9):2987-3000. [English].

7. Eken G, Misir A, Demirag B, Ulusaloglu C, Kizkapan TB. Delayed or neglected meniscus tear repair and meniscectomy in addition to ACL reconstruction have similar clinical outcome. Knee surgery, sports traumatology, arthroscopy : official journal of the ESSKA 2020;28(11):3511-16. [English].

8. Hatayama K, Terauchi M, Saito K, Takase R, Higuchi H. Healing Status of Meniscal Ramp Lesion Affects Anterior Knee Stability After ACL Reconstruction. Orthop J Sports Med 2020;8(5):2325967120917674. [eng].

9. Hoshino T, Nakagawa Y, Inomata K, Ohara T, Katagiri H, Otabe K, et al. Effects of different surgical procedures for meniscus injury on two-year clinical and radiological outcomes after anterior cruciate ligament reconstructions. -TMDU MAKS study. Journal of Orthopaedic Science 2021. [English].

10. Kacmaz IE, Gezer MC, Basa CD, Zhamilov V, Ekizoglu O. Use of the forgotten joint score (FJS)-12 to evaluate knee awareness after isolated anterior cruciate ligament reconstruction with and without meniscus repair or partial meniscectomy. European Journal of Orthopaedic Surgery and Traumatology 2021. [English].

11. LaPrade CM, Dornan GJ, Granan LP, LaPrade RF, Engebretsen L. Outcomes After Anterior Cruciate Ligament Reconstruction Using the Norwegian Knee Ligament Registry of 4691 Patients: How Does Meniscal Repair or Resection Affect Short-term Outcomes? The American journal of sports medicine 2015;43(7):1591-97. [English].

12. Lee WQ, Gan JZ, Lie DTT. Save the meniscus - Clinical outcomes of meniscectomy versus meniscal repair. J Orthop Surg (Hong Kong) 2019;27(2):2309499019849813. [eng].

13. Lepley LK, Wojtys EM, Palmieri-Smith RM. Does concomitant meniscectomy or meniscal repair affect the recovery of quadriceps function post-ACL reconstruction? Knee surgery, sports traumatology, arthroscopy : official journal of the ESSKA 2015;23(9):2756-61. [English].

14. Melton JTK, Murray JR, Karim A, Pandit H, Wandless F, Thomas NP. Meniscal repair in anterior cruciate ligament reconstruction: A long-term outcome study. Knee Surgery, Sports Traumatology, Arthroscopy 2011;19(10):1729-34. [English].

15. Michalitsis S, Hantes M, Thriskos P, Tsezou A, Malizos KN, Fezoulidis I, et al. Articular cartilage status 2 years after arthroscopic ACL reconstruction in patients with or without concomitant meniscal surgery: evaluation with 3.0T MR imaging. Knee surgery, sports traumatology, arthroscopy : official journal of the ESSKA 2017;25(2):437-44. [English].

16. Phillips M, Rönnblad E, Lopez-Rengstig L, Svantesson E, Stålman A, Eriksson K, et al. Meniscus repair with simultaneous ACL reconstruction demonstrated similar clinical outcomes as isolated ACL repair: a result not seen with meniscus resection. Knee surgery, sports traumatology, arthroscopy : official journal of the ESSKA 2018;26(8):2270-77. [English].

17. Shelbourne KD, Carr DR. Meniscal repair compared with meniscectomy for bucket-handle medial meniscal tears in anterior cruciate ligament-reconstructed knees. American Journal of Sports Medicine 2003;31(5):718-23. [English].

18. Shelbourne KD, Dersam MD. Comparison of partial meniscectomy versus meniscus repair for bucket-handle lateral meniscus tears in anterior cruciate ligament reconstructed knees. Arthroscopy - Journal of Arthroscopic and Related Surgery 2004;20(6):581-85. [English].

19. Singh A, Wei DT, Lin CTP, Liang S, Goyal S, Tan KA, et al. Concomitant meniscal injury in anterior cruciate ligament reconstruction does not lead to poorer short-term post-operative outcomes. Knee surgery, sports traumatology, arthroscopy : official journal of the ESSKA 2018;26(4):1266-72. [English].

20. Svantesson E, Cristiani R, Hamrin Senorski E, Forssblad M, Samuelsson K, Stålman A. Meniscal repair results in inferior short-term outcomes compared with meniscal resection: a cohort study of 6398 patients with primary anterior cruciate ligament reconstruction. Knee surgery, sports traumatology, arthroscopy : official journal of the ESSKA 2018;26(8):2251-58. [English].

21. Wang M, Lin Z, Wang W, Chen L, Xia H, Zhang Y, et al. Kinematic Alterations After Anterior Cruciate Ligament Reconstruction via Transtibial Techniques With Medial Meniscal Repair Versus Partial Medial Meniscectomy. Am J Sports Med 2021;49(12):3293-301. [eng].

22. Yang YP, Ma X, An H, Liu XP, An N, Ao YF. Meniscus repair with simultaneous anterior cruciate ligament reconstruction: Clinical outcomes, failure rates and subsequent processing. Chinese Journal of Traumatology - English Edition 2021. [English].
